# Application of human amniotic epithelial cells in regenerative medicine: a systematic review

**DOI:** 10.1186/s13287-020-01951-w

**Published:** 2020-10-15

**Authors:** Qiuwan Zhang, Dongmei Lai

**Affiliations:** The International Peace Maternity and Child Health Hospital, School of Medicine, Shanghai Jiao Tong University; Shanghai Key Laboratory of Embryo Original Diseases; Shanghai Municipal Key Clinical Speciality, 145, Guang-Yuan Road, Shanghai, 200030 People’s Republic of China

**Keywords:** Human amniotic/amnion epithelial cells, Cell transplantation, Differentiation, Paracrine properties, Immunomodulation, Regenerative medicine

## Abstract

Human amniotic epithelial cells (hAECs) derived from placental tissues have gained considerable attention in the field of regenerative medicine. hAECs possess embryonic stem cell-like proliferation and differentiation capabilities, and adult stem cell-like immunomodulatory properties. Compared with other types of stem cell, hAECs have special advantages, including easy isolation, plentiful numbers, the obviation of ethical debates, and non-immunogenic and non-tumorigenic properties. During the past two decades, the therapeutic potential of hAECs for treatment of various diseases has been extensively investigated. Accumulating evidence has demonstrated that hAEC transplantation helps to repair and rebuild the function of damaged tissues and organs by different molecular mechanisms. This systematic review focused on summarizing the biological characteristics of hAECs, therapeutic applications, and recent advances in treating various tissue injuries and disorders. Relevant studies published in English from 2000 to 2020 describing the role of hAECs in diseases and phenotypes were comprehensively sought out using PubMed, MEDLINE, and Google Scholar. According to the research content, we described the major hAEC characteristics, including induced differentiation plasticity, homing and differentiation, paracrine function, and immunomodulatory properties. We also summarized the current status of clinical research and discussed the prospects of hAEC-based transplantation therapies. In this review, we provide a comprehensive understanding of the therapeutic potential of hAECs, including their use for cell replacement therapy as well as secreted cytokine and exosome biotherapy. Moreover, we showed that the powerful immune-regulatory function of hAECs reveals even more possibilities for their application in the treatment of immune-related diseases. In the future, establishing the optimal culture procedure, achieving precise and accurate treatment, and enhancing the therapeutic potential by utilizing appropriate preconditioning and/or biomaterials would be new challenges for further investigation.

## Background

Human amniotic/amnion epithelial cells (hAECs) are derived from the innermost layer of the term placenta closest to the fetus, and they have been shown to have the potential to be seed cells for allogeneic cell therapies. Over the past 20 years, interest has been growing regarding the utility of hAECs in regenerative medicine due to their proliferative capacity, multilineage differentiation potential, ease of access, and safety. Advances in stem cell-based approaches have revealed that hAECs are perinatal stem cells that possess embryonic stem cell-like properties and the ability to be induced to differentiate. As promising seed stem cell, hAECs have been widely used to treat various diseases through transplantation therapy. Evidence supported by animal studies has revealed that hAECs show therapeutic potential for treatment of many diseases, including neurological disorders [1–4], lung injury [5, 6], liver injury [7], diabetes [8], acute kidney failure [9], cardiovascular diseases [10], wound healing [11], healing of stage III pressure ulcers [12], intrauterine adhesion [13], and premature ovarian failure (POF) [14]. Although hAECs have exhibited good therapeutic efficacy, they possess differences in differentiation potential, secretory function, and immunomodulatory activity under different conditions, producing specific effects depending on their application. In this review, we mainly examined the literature about the therapeutic potential of hAECs and summarized the different repair mechanisms in injured tissues and disorders; we also discussed the induced differentiation plasticity, homing and differentiation, paracrine function, and immunomodulatory capacity of hAECs. Finally, we described the current research strategies and proposed new prospects for hAEC-based clinical applications in the future.

## Methods

Comprehensive literature searches using PubMed, MEDLINE, or Google Scholar were performed to identify articles for review written in English focusing on the biology of hAECs and the role of hAECs in injured tissues, diseases, and regenerative medicine.

### Biological characteristics of hAECs

hAECs are obtained from the amniotic membrane of term placentas, which are discarded after birth. Thus, hAECs are readily available, require no invasive procedures for harvesting, and avoid relevant ethical issues. Isolated hAECs express surface markers of embryonic stem cells such as stage-specific embryonic antigen-3 and 4 (SSEA-3 and SSEA-4), octamer-binding transcription factor-4 (OCT-4), and tumor rejection antigen1-60 and 1-80 (TRA1-60 and TRA1-80) [15]. Although hAECs possess stem cell-like characteristics, they do not exhibit unlimited proliferation due to the lack of telomerase activity and no risk of tumorigenesis in vivo [16]. Moreover, hAECs have tri-mesodermal lineage differentiation potential; they can produce osteogenic, adipogenic, and chondrogenic lineages under appropriate culture conditions [17]. Notably, hAECs have low expression levels of human leukocyte antigens (including HLA-A, HLA-B, and HLA-C and HLA-DR), which are key antigens involved in recipient rejection [1, 18]. A study confirmed that intravenous administration of hAECs did not result in hemolysis, allergic reactions, toxicity, or tumor formation, which demonstrated that hAECs were systematically safe [19]. Thus, hAECs are considered a promising source of stem cells for regenerative medicine.

To safely and effectively use hAECs to repair the function of injured tissues and treat diseases, it is essential that they are of high quality. Currently, the trypsin/EDTA technique is the most efficient for obtaining viable hAECs for subsequent culture [20, 21]. The key aspects involved in the isolation of hAECs with high yield, viability, and purity have been previously summarized [22]. Meanwhile, standard manufacturing and cryopreservation process were established that resulted in the isolation of highly purified hAECs with reproducible and high viability in accordance with current good manufacturing procedure (GMP) requirements [23]. In addition, a study found that epidermal growth factor (EGF) was a strong mitogen that promoted the proliferation of hAECs by regulating the cell cycle rather than inducing differentiation in the process of culture in vitro [24]. Although primary hAECs strongly expressed stemness-related genes for OCT-4, Sox-2, and Nanog, these gene expression levels gradually declined with an increase in passage number [25]. Study revealed that cultured hAECs underwent epithelial to mesenchymal transition (EMT) through the autocrine production of TGF-beta (TGF-β) [26]. Moreover, TNF-alpha (TNF-α) and matrix metalloproteinase (MMP) could induce EMT of hAECs [27]. Therefore, researchers have tried to explore different culture methods to maintain stemness and avoid EMT occurrence. A study found that hAECs were cultured in substitute serum medium (SSM) in which fetal calf serum (FCS) was replaced by knockout serum replacement (SR), contributing to maintain stem cell characteristics for up to 4 passages [28]. Furthermore, xenobiotic-free medium was used for the culture of hAECs to eliminate the effects of growth factors [29]. Meanwhile, a serum-free protocol established for hAEC isolation and culture resulted in better cell growth than that achieved by a traditional culture system with serum [19], which made the clinical applications of hAECs more feasible.

Although the biological characteristics of hAECs could be affected by the culture conditions and number of passages, they can be sufficiently expanded under certain culture conditions and maintain reproducible biological characteristics. Therefore, further understanding of the biological characteristics of hAECs and improvement of the isolation and culture techniques are important for applying hAECs in regenerative medicine.

### Induced differentiation plasticity of hAECs in vitro

One study indicated that hAECs were more efficiently reprogrammed to assume a state of induced pluripotency than adult fibroblasts [30]. An increasing number of studies have found that hAECs display an extremely high level of differentiation plasticity in vitro following chemical induction, biological treatment, gene transfection, or coculture with other types of cells (Table 1).

**Table 1:** The induced differentiation of hAECs in vitro

Organ/focuses	Cell types	Phenotypes	Inducing conditions	References
**Liver**	Hepatocyte-like cells	Expressing hepatocyte-like cell functional genes: albumin, CYP1A1, CYP1A2, c-met, and transcription factors: HNF3, HNF4, C/EBPa, and HNF1	Using a combined approach of dexamethasone, HGF, IGF, and other cytokines	[31]
	Hepatocyte-like cells	Expressing hepatic related genes: albumin, A1AT, CYP3A4, 3A7, 1A2, 2B6, and the asialoglycoprotein receptor 1 (ASGPR1)	Using extracellular matrix substrates; cocultured with mouse hepatocytes	[32]
	Hepatic differentiation	Displayed a similar hepatic morphology; expressing specific hepatic genes: albumin, CYP7A1, and CYP3A4	Using a specific hepatic differentiation protocol	[33]
	Hepatic differentiation	The formation of bile canaliculi; secreting albumin; uptaking low-density lipoprotein and showing inducible CYP3A4 and CYP2C9 enzymatic activities	Using four-step hepatic differentiation protocol	[34]
	Hepatic sinusoidal endothelial cells	Forming capillary-like structure in vitro and differentiate into HSECs in vivo	Under proangiogenic conditions	[35]
**Pancreas**	Insulin-producing cells	The formation of three-dimensional (3D) spheroids; producing pancreatic endocrine hormones; releasing C-peptide under hyperglycemic condition	Culturing on extracellular matrix	[36]
	Pancreatic lineage cells	Expressing pancreatic endoderm and progenitor genes: NKX6.1, NeuroD1, and pancreatic lineage genes: PDX1, SOX17, RFX6	Combination of transcription factor PDX1 with activin A or nicotinamide	[37]
	Islet-like cells	Expressing the endocrine-related genes: PDX1, ngn3, insulin, and glucagon; secreting insulin in response to high glucose exposure	Using DMEM with different supplements and suspension culture	[38]
	Islet-like cell clusters	Expressing pancreatic development-related genes: PDX1, NKX6-1, NEUROG3, PAX6, INS, and GCG; insulin positive and sensitive to glucose	Adding nicotinamide plus betacellulin	[39]
	Pancreatic cells	Expressing pancreatic differentiation related genes: NKX6.1, SOX17, RFX6, NEUROD1, and PAX4	Inducing endogenous PDX1 expression, EGF, and poly-l-ornithine	[40]
	Acinar cells	Expressing α-amylase and mucins	Cocultured with submandibular gland acinar cells using a double-chamber system	[41]
	Insulin secreting cells	Expressing PDX1 and beta2 microglobulin; secreting insulin	Treated with nicotinamide and N2 supplement	[42]
**Ovary**	Germ cell-like cells	Expressing germ cell-specific genes: GDF9, DAZL, and SCP3; producing estradiol	Medium supplemented with 5% human follicular fluid	[43]
	Follicle-like structure	Expressing germ cell-specific genes DAZL and GDF9; secreting estradiol	Medium supplemented with 5% human follicular fluid	[44]
	Germ cell-like cells (diploid cells)	Expressing germ cell-specific protein DAZL, oocyte-specific proteins GDF9 and ZP3, meiosis-specific proteins DMC1 and SCP3	Cultured in medium containing serum substitute supplement (SSS)	[45]
**Eyes**	Corneal epithelial-like cells	Showing a similar morphology to hCECs; expressing CK3/12, CK14, CK19, and P63	Cultured with human corneal epithelial cells (hCECs) in a Transwell coculture system	[46]
	Corneal epithelial-like cells	Expressing CK3/12	Seeded onto rabbit corneal stroma	[47]
	Corneal epithelial-like cells	Expressing CK3/12	Adding the conditioned medium of spontaneously immortalized human corneal epithelial cells (S-ihCECs)	[48]
	Conjunctival epithelium-like cells	Showed conjunctival epithelium phenotype; producing mu5ac	Cultured with induced-denuded conjunctival matrix and conjunctival homogenate	[49]
**Nervous system**	Neuronal differentiation	Expressing neural cell markers NSE and NeuN	Adding noggin, bFGF, and retinoic acid	[50]
	Neuronal differentiation	Upregulation of transcription factors involving in neuronal differentiation	Treated with rosmarinic acid	[51]
	Cortical progenitors	Expressing cortical neuron-specific proteins: TBR2, OTX2, NeuN, and β-III-tubulin	Adding growth factors and small molecules	[52]
	Schwann-like cells	Exhibiting a typical bipolar or tripolar morphology; expressing S-100; increasing the expressions of BDNF and GDNF	Cocultured with Schwann cells (SCs)	[53]
**Bone**	Osteogenic differentiation	Increasing cellular ALP activity and extracellular mineralization; expressing Runx2, osterix, ALP, collagen I, and OPN	Cultured with classic osteogenic medium	[54]
	Cartilage differentiation	Expressing cartilage markers: aggrecan, Sox9, CEP-68, and type II and X collagens; promoting matrix synthesis	Treated with BMP-7 or TGF-β1	[55]
	Osteoblasts	Upregulating Runx2, ALP, and OPN	Mechanical stretch	[56]
**Respiratory**	Polarized airway-like cells	Forming 3D structures; expressing CFTR and possessing functional iodide/chloride (I^−^/Cl^−^) ion channels	Cultured with small airway growth medium (SAGM)	[57]
**Heart**	Cardiomyocyte-like cells	Expressing cardiac-specific genes Nkx2.5 and alpha-actinin	Treated with activin A and BMP-4	[58]
**Skin**	Epidermal cells	The presence of desmosomes; expressing CK18 and CK14	Cultured in air-liquid interface	[59]

A series of studies reported that hAECs were successfully induced to differentiate into hepatocyte-like cells through a combined approach using dexamethasone, hepatocyte growth factor (HGF), insulin-like growth factor (IGF), and other cytokines [31]; extracellular matrix proteins together with a mixture of growth factors, cytokines, and hormones or cocultured with muse hepatocytes [32]; a specific hepatic differentiation protocol [33]; and four-step hepatic differentiation [34]. In addition, hAECs could respond to proangiogenic signals, form capillary-like structures, and differentiate into hepatic sinusoidal endothelial cells (HSECs) in vivo [35].

With regard to pancreatic cells, hAECs were induced to form three-dimensional (3D) spheroids and differentiate into insulin-producing cells by culturing them on an extracellular matrix [36]. When hAECs were treated with activin A or nicotinamide combined with the transcription factor pancreatic and duodenal homeobox-1 (PDX1), they differentiated into pancreatic lineage cells [37]. Furthermore, hAECs differentiated into islet-like cells expressing endocrine-related genes, including PDX1, insulin, and glucagon, as a result of the addition of nicotinamide plus betacellulin; importantly, the differentiated islet-like cells secreted high levels of insulin in response to high glucose exposure [38, 39]. In addition, the potential for the differentiation of hAECs into pancreatic cells triggered by overexpressing PDX1 could be strengthened with a mix of EGF and poly-l-ornithine in the culture environment [40]. A study showed that hAECs cocultured with submandibular gland acinar cells in a double-chamber system differentiated into acinar cells [41]. When hAECs were treated with nicotinamide and N2 supplement, they differentiated into insulin secreting cells (ISCs) expressing PDX1 and beta2 microglobulin, meaning that they show potential for application to cell therapy of type I diabetes [42].

The fluid from specific tissues and organs could also affect the process of hAEC differentiation. It was confirmed that hAECs cultured with medium containing 5% human follicular fluid expressed the germ cell-specific markers GDF9 and DAZL [43], and formed a follicle-like structure [44]. By using serum substitute supplement (SSS) medium, hAECs were induced to differentiate into germ cells expressing DAZL, VASA, GDF9, and ZP3 [45].

The ability of hAECs to differentiate into corneal epithelial-like cells has been confirmed in many studies. For example, hAECs were induced to differentiate into corneal epithelial-like cells when they were cocultured with human corneal epithelial cells (hCECs) [46] and seeded onto rabbit corneal stroma [47] or by using culture media collected from spontaneously immortalized human corneal epithelial cells (S-ihCECs) to replicate the corneal epithelial microenvironment [48]. These methods may be suitable for the reconstruction of the corneal epithelium. Additionally, hAECs were able to differentiate into conjunctival epithelium-like cells with partial physiological function upon culture with induced-denuded conjunctival matrix and conjunctival homogenate [49].

The capability of hAECs to differentiate into neural cells was affected by factors including serum, noggin, basic fibroblast growth factor (bFGF), and retinoic acid [50]. A recent study showed that hAECs were induced to undergo neuronal differentiation by treatment with rosmarinic acid [51]. hAECs differentiated into cortical progenitor lineage cells and showed a cortical neuron phenotype when treated with growth factors and small molecules [52]. Additionally, hAECs were induced to differentiate into Schwann-like cells (SCs) that exhibited the morphological, phenotypic, and functional characteristics of SCs when they were treated according to a coculture approach [53].

Osteogenic differentiation of hAECs was induced by the upregulation of Runx2, osterix, alkaline phosphatase (ALP), collagen I, and osteopontin (OPN) in vitro [54]. hAECs treated with either bone morphogenetic protein (BMP)-7 or TGF-β1 expressed cartilage markers, including aggrecan, Sox-9, CEP-68, and type II and X collagens [55]. Interestingly, the osteogenic differentiation of hAECs was also induced by mechanical stretching [56], which means that changing the physical conditions may be a new approach to affect the process of hAEC differentiation.

Additionally, hAECs could form 3D structures and express the cystic fibrosis transmembrane conductance regulator (CFTR) by adding small airway growth medium (SAGM) [57]. When treated with activin A and BMP-4, hAECs were able to express cardiac-specific genes, including Nkx2.5 and alpha-actinin, indicating that hAECs have the potential to differentiate into cardiomyocytes [58]. In addition, the air-liquid interface could stimulate the early differentiation of hAECs into epidermal cells, which indicates their potential use for skin regeneration [59].

Taken together, these studies indicate that hAECs have strong potential to be induced to differentiate via changes in culture conditions and methods. Inducing the differentiation of hAECs toward a desired phenotype in vitro before injection or transplantation will be an effective method for replacing damaged cells for tissue regeneration.

### Homing and differentiation of hAECs in vivo

In addition to culture conditions, the differentiation potential of hAECs largely depends on the specific organizational microenvironment in vivo following transplantation. Grafted hAECs could migrate to injured tissue and further differentiate into the appropriate cell type under different conditions. Extensive animal studies have confirmed the capacity of hAECs to differentiate into essential and specialized cell types, which participate in the functional recovery of damaged tissues (Table 2).

**Table 2:** Homing and differentiation of hAECs in vivo

Diseases/focuses	Transplantation method/dose	Species	Outcome	Repair mechanism	References
Parkinson’s disease	Injection of striatum (4 × 10^4^ cells)	Rats	Ameliorating of apomorphine-induced rotational asymmetry	Differentiating into TH-immunoreactive cells	[60]
Ischemic brain injury	Injection of dorsolateral striatum (8 × 10^5^ cells)	Rats	Ameliorating behavioral dysfunction; reducing infarct volume	Expressing neuronal progenitor marker (Nestin), neuronal marker (MAP 2), astrocyte marker (GFAP)	[61]
Alzheimer’s disease	Intracerebroventricular injection (1.2 × 10^5^ cells)	Mice	Improving the spatial memory; increasing acetylcholine concentration and the number of hippocampal cholinergic neurites	Expressing stem cell-specific markers OCT-4 and Nanog	[62]
Chronic liver failure	Intrasplenical injection (2 × 10^6^ cells)	Mice	Liver was larger in size, softer, and less nodular; increasing serum albumin level	Differentiating into functional hepatocytes positive for albumin	[34]
Lung injury	Fetal jugular vein (3 × 10^6^ cells)	Sheep	Reducing ventilation-induced preterm lung injury, including less collagen, elastin, fibrosis, normalized secondary-septal crests	Differentiating into type I and II alveolar cells	[63]
Premature ovarian failure	Intravenously injection (2 × 10^6^ cells)	Mice	Promoting folliculogenesis; repairing ovarian function	Differentiating into granulosa cells expressing follicle-stimulating hormone receptor (FSHR)	[64]
Myocardial infarction	Injection of the infarcted myocardium (1 × 10^6^ cells)	Rats	Decreasing infarct size; improving cardiac function	Differentiating into cardiomyocyte-like cells expressing myocardium-specific marker myosin heavy chain	[65]
Gland injury	Intra-glandular injection (1 × 10^6^ cells)	Mice	Restoring the morphology and function of salivary gland	Differentiating into acinar-like cells	[66]
Inner ear injury	Injection of cochlea (1 × 10^5^ cells)	Hartley guinea pigs	Cooperation in the regional potassium ion recycling	Expressing cochlear fibrocyte markers connexin 26 and Na-K-adenosine triphosphatase	[67]
Achilles tendon injury	In situ injection (10 × 10^6^ cells)	Sheep	Improving tendon microarchitecture and blood vessel remodeling; contributing to tendon regeneration	Differentiating into tenocytes expressing collagen I	[68]

Generally, the initial presence of injury or damage causes early cell death and loss of functional cells, and then a series of secondary reactions occur, including hypoxia, inflammation, ischemia, and dysfunction. Study reported that hAECs not only expressed specific markers of nerve cells, but also migrated along nerve fibers in the corpus callosum [69]. A series of studies demonstrated that hAECs exerted neuroprotective effects, possibly in relation to neuronal differentiation. In a Parkinson’s disease (PD) model, hAECs migrated to and survived in the injured striatum, and partially ameliorated apomorphine-induced rotational asymmetry through differentiating into TH-immunoreactive cells [60]. Another study found that grafted hAECs migrated to the injured brain area via a CXC chemokine receptor type 4 (CXCR4)-dependent mechanism in ischemic stroke [70], and chemokines released by damaged tissues were a key mediator of transplanted stem cell tracking to the site of injury [71]. In ischemic brain injury, grafted hAECs migrated into the ischemic area and expressed the neuronal specific marker (MAP 2) and neuronal progenitor marker (Nestin), and they significantly ameliorated behavioral dysfunction and reduced infarct volume [61]. In transgenic mice with Alzheimer’s disease (AD), transplanted hAECs survived for at least 8 weeks, and they were shown to migrate to the third ventricle without immune rejection and to express the stem cell markers OCT-4 and Nanog [62]. In thioacetamide-induced chronic liver failure, engrafted hAECs migrated into the liver and differentiated into functional hepatocyte-like cells, improved the state of the liver following chronic injury, and produced a high level of serum albumin [34]. In addition, transplanted hAECs differentiated into type I and II alveolar cells and mitigated ventilation-induced preterm lung injury [63]. In chemotherapy-induced POF, grafted hAECs were shown to migrate to the injured ovaries and differentiate into granulosa cells to restore folliculogenesis and ovarian function [64]. In myocardial infarction, hAECs improved cardiac function following transplantation by differentiating into cardiomyocyte-like cells [65]. In gland injury induced by radiation, hAECs in the recipient glands differentiated into acinar-like cells, resulting in morphological and functional restoration of the salivary gland [66]. Additionally, transplanted hAECs could express connexin 26 and Na-K-adenosine triphosphatase in the inner ear [67]. In Achilles tendon injury, hAECs remained viable within the host tendons and established an active dialogue with endogenous progenitor cells, and the differentiated hAECs displayed a tenocyte-like phenotype and contributed to the recovery of the function of Achilles tendon [68].

Although numerous studies have demonstrated that hAECs can migrate and differentiate into the desired type of cells to replace damaged cells, only a few studies have reported the long-term functional integration of engrafted hAECs in target organs. The limited homing efficiency and the lack of long-term cell tracking approaches could be major reasons for the lack of functional research on differentiated cells. In addition to the efficacy of engraftment, several additional factors for hAEC therapy need to be considered, including the number of administered cells, the route of infusion, and the biodistribution of cells post-transplantation. Although tail vein injection is the most widely used transplantation method to deliver cells into a host, most engrafted cells are trapped in the lungs rather than the target organs. For example, splenic injection was a more efficient route of administration of hAECs for targeting the liver than tail vein infusion [72, 73]. Therefore, selecting an appropriate transplantation route is vital for cell survival, differentiation of grafted cells, and the recovery of function.

Therefore, transplanted hAECs can be recruited to damaged tissues and exert differentiation plasticity in vivo based on the specific types of cells needed for replacement therapy to treat many diseases.

### Paracrine potential of hAECs

Migration of grafted hAECs to injured tissues and replacement of damaged cells are thought to be the mechanisms behind the alleviation of acute and chronic injury; however, there are several obstacles to cell transplantation, such as poor survival and limited restoration ability. Currently, accumulating evidence has demonstrated that hAECs can provide a beneficial microenvironment for cell survival and activate endogenous mechanisms of tissue regeneration by secreting bioactive cytokines and microvesicles. This is supported by evidence that the injection of the conditioned media of hAECs (hAEC-CM) could achieve a positive outcome similar to that of cell transplantation, representing an acceptable alternative for stem cell-free biotherapy. As early as 2000, a study showed that hAECs could synthesize and release brain-derived neurotrophic factor (BDNF), neurotrophin-3 (NT-3), and nerve growth factor (NGF), which play important roles in the early stages of neural development in the embryo as well as the neuroprotective effect [74, 75]. Moreover, study also showed that hAECs secreted considerable amounts of proangiogenic, anti-fibrotic, and anti-inflammatory factors, including basic fibroblast growth factor (bFGF), vascular endothelial growth factor (VEGF), angiogenin (AGN), and insulin-like growth factor (IGF) [76]. The therapeutic function of these bioactive secretory factors in various disease models has been studied and confirmed (Table 3).

**Table 3:** Paracrine function of hAECs in different diseases

Diseases/focuses	Injection method	Species	Outcome	Repair mechanism	References
Parkinson’s disease	Injection of tegmentum of the midbrain	Rats	Enhancing the survival of DA; protecting the morphological integrity of TH-positive neurons against toxic insult	hAEC-CM (neurotrophins such as BDNF and NT-3)	[77]
Corneal alkali injury	Injection the dorsal bulbar subconjunctival	Rabbits	Reducing the infiltration of inflammatory cells; promoting corneal wound healing	hAEC-CM (anti-inflammatory factors)	[78]
Corneal injury	Topically application	Mice	Reducing corneal neovascularization; suppressing corneal inflammatory reactions	hAEC-CM (anti-inflammatory factors)	[79]
Premature ovarian failure	Intraperitoneally injection	Mice	Promoting the formation of vascular; restoring ovarian function	hAEC-CM (proangiogenic factors)	[80]
Premature ovarian failure	Ovarian injection	Mice	Promoting follicular development; inhibiting granulosa cell apoptosis; restoring ovarian function	hAEC-CM (TGF-β1; anti-apoptotic effect)	[81]
Chronic liver fibrosis	Intravenously injection	Mice	Reducing collagen synthesis and macrophage infiltration; inducing macrophage toward M2 phenotype	hAEC-CM (anti-fibrosis, anti-inflammation)	[82]
Non-alcoholic steatohepatitis	Intraperitoneally injection	Mice	Reducing hepatic inflammation; inhibiting liver fibrosis	hAEC-CM (anti-inflammation; anti-fibrosis)	[83]
Myocardial infraction	Cardiac injection	Rats	Regenerating myocardial tissue; improving cardiac function	hAEC-secreting proangiogenic factors	[10]
Wound healing	Topically injection	Rats	Promoting the migration and proliferation of fibroblasts; accelerating wound healing; inhibiting scar formation	hAEC-exosomes	[84, 85]
Wound healing	Topically injection	Mice	Stimulating the migration and proliferation of fibroblasts; accelerating wound healing	hAEC-exosomes (miRNAs)	[86]
Idiopathic pulmonary fibrosis	Intravenously injection; intranasal instillation	Mice	Reducing lung inflammation and fibrosis; improving tissue-to-airspace ratio	hAEC-exosomes (anti-inflammation; anti-fibrosis)	[87]
Chronic liver fibrosis	Intravenously injection	Mice	Reducing collagen synthesis and macrophage infiltration; inducing macrophage toward to M2 phenotype	hAEC-EV (anti-fibrotic proteins)	[82]
Premature ovarian failure	Ovarian injection	Mice	Inhibiting the apoptosis of granulosa cells; repairing ovarian function	hAEC-exosomes (miR-1246; anti-apoptosis)	[88]

For example, hAECs enhanced the survival of dopamine (DA) neurons by producing biologically activated neurotrophins, such as BDNF and NT-3, and they counteracted the loss of DA neurons in PD model mice [60, 77]. In corneal injury, hAEC-CM injection promoted corneal wound healing by reducing the infiltration of inflammatory cells [78] and reducing corneal neovascularization [79]. hAEC-CM injection protected ovaries against chemotherapy-induced damage, enhanced the ovarian microenvironment, and promoted ovarian vessel formation [80]. Further study found that hAEC-secreting TGF-β1 played important roles in protecting granulosa cells against apoptosis [81]. On the other hand, study has shown that hAECs suppressed collagen production in human hepatic stellate cells through the paracrine pathway [89], and some important soluble factors such as MMP-2 and MMP-9 secreted by hAECs played vital roles in anti-fibrosis and promoting extracellular matrix (ECM) remodeling, especially in liver injury and fibrosis models [7, 82]. In a diet-induced murine non-alcoholic steatohepatitis (NASH) model, administration of hAEC-CM reduced liver fibrosis and hepatic inflammation, supporting the anti-fibrotic properties of hAEC-CM [83]. Although hAECs have been reported to secrete angiogenic factors, including EGF, VEGF, AGN, and platelet-derived growth factor B (PDGFB), there have been some contradictory reports on their angiogenic effects to date. A study revealed that significantly different protective effects were exerted by term and preterm hAECs in vivo [90], which was related to the differences in the angiogenic ability of hAECs isolated from different gestational stages [91]. In addition, the impact of hAECs on angiogenesis could be influenced by the presence of inflammation in injured tissue [91]. Study found that hAECs under hypoxic condition could secrete the high levels of the human-origin proangiogenic cytokines, contributing to myocardial tissue regeneration [10]. Thus, the impact of microenvironmental biological cues on the paracrine function of transplanted hAECs should be considered to identify the optimal times for cell administration.

In addition, microvesicles or exosomes, as essential components of the hAEC paracrine pathway, have attracted much attention. Exosomes are small secretory vesicles that are involved in intercellular communication via the transport of bioactive cargo, including proteins, mRNA, microRNAs (miRNAs), and organelles [92]. Importantly, study has demonstrated that exosomes released by stem cells can effectively transport proteins, mRNAs, and miRNAs to exert a variety of effects on target tissues [93]. It has been shown that hAEC-derived exosomes (hAEC-exosomes) promoted the migration and proliferation of fibroblasts, downregulated collagen expression, and improved skin wound healing by inducing the formation of well-organized collagen fibers in rats [84, 85]. Further study indicated that the effects of hAEC-exosomes were abolished by pretreating hAEC-exosomes with RNaseA, which indicates that miRNAs carried by exosomes play important roles in promoting wound healing [86]. In bleomycin-induced lung injury, hAEC-exosome transplantation reduced inflammation and fibrosis, and improved the tissue-to-airspace ratio by increasing macrophage phagocytosis, reducing neutrophil myeloperoxidase activity, and directly suppressing T cell proliferation. Further analysis found that some specific proteins comprising the cargo of hAEC-exosomes were mainly enriched in the MAPK signaling, apoptotic, and developmental biology pathways; however, the miRNAs were enriched in the PI3K-Akt, Ras, Hippo, TGF-β, and focal adhesion pathways [87]. Moreover, study also showed that proteins in hAEC-derived extracellular vesicles (hAEC-EVs) exerted anti-fibrosis function via modulating collagen synthesis and macrophage polarization in chronic liver fibrosis [82]. In addition, the target genes of miRNAs in hAEC-exosomes were mainly enriched in the phosphatidylinositol signaling system, PPAR signaling pathway, and apoptotic process pathway, which are involved in ovarian functional recovery mediated by hAEC-exosome transplantation [88].

Taken together, these results demonstrated that hAEC-derived cytokines and exosomes have important repair potential in the treatment of various diseases, imitating cell transplantation and avoiding the shortcomings of stem cell transplantation. Thus, exploring and identifying the most effective secretome components, including bioactive factors and extracellular vesicles/exosomes secreted by hAECs, will provide new treatment strategies for regenerative medicine.

### Immunomodulatory properties of hAECs

In addition to the differentiation ability and paracrine function of hAECs, their powerful immunomodulatory properties also make their use in regenerative medicine a more reasonable option than the use of other cell types. Many studies have demonstrated that hAECs and hAEC-CM exert multiple immunosuppressive activities and anti-inflammatory properties. For example, hAECs suppressed both specific and non-specific T cell proliferation, decreased pro-inflammatory cytokine production, and inhibited the activation of stimulated T cells in vitro [94]. Furthermore, hAECs prevented monocyte-derived dendritic cell differentiation via cell-to-cell contact [95]. In addition, hAECs could produce a variety of immunoregulatory factors, including migration inhibitory factor (MIF), TGF-β, interleukin-10 (IL-10), and prostaglandin E2 (PGE2), contributing to suppress the function of inflammatory cells [96]. When hAECs were cocultured with peripheral blood mononuclear cells (PBMCs) derived from patients with unexplained recurrent spontaneous abortion, the proliferation of naïve CD4 T cells was significantly inhibited, and the production of Th1 and Th17 cytokines was reduced [97, 98]. hAEC significantly attenuated the level of oxidative burst of neutrophils in coculture system [99]. Additionally, hAEC-CM inhibited the chemotactic activity of neutrophils and reduced the proliferation of both T and B cells after mitogenic stimulation [100].

These immunomodulatory properties have laid the foundation for the use of these cells in treating inflammatory and immune-based diseases, and encouraging results have been obtained in different disease models (Table 4). In ischemic stroke, hAEC transplantation significantly reduced inflammation, leading to the recovery of brain function [70] and the improvement of brain function after intracerebral hemorrhage (ICH) by reducing microglial activation and producing anti-inflammatory factors [101]. In perinatal brain injury, hAEC transplantation reduced apoptosis and astrocyte areal coverage in the white matter, and increased the density of total and activated microglia via the release of trophic factors [102]. Moreover, hAECs mitigated fetal brain inflammation and reduced white matter injury via anti-inflammatory effects in the preterm ovine fetus, and they reduced the number of activated microglial cells in the white matter after repeated endotoxin exposure [103] and lipopolysaccharide (LPS)-induced intrauterine inflammation [104]. In multiple sclerosis, splenocytes from hAEC-treated mice showed a Th2 cytokine shift with significantly elevated interleukin-5 (IL-5) production [105]. In lung injury, a series of studies demonstrated that transplanted hAECs repaired lung function by decreasing macrophage and neutrophil infiltration, fibrosis, and collagen content; importantly, hAECs required normal host macrophage function to exert their reparative effects [106]. Further study showed that hAECs mediated lung function recovery by modulating macrophage recruitment and polarization in a paracrine manner [107]. Moreover, hAECs induced the maturation of non-Tregs into FoxP3-expressing Tregs mediated by TGF-β. Tregs are required for hAEC-mediated macrophage polarization and lung function recovery [108]. Another study also showed that the immunomodulatory effects of hAECs on macrophage phagocytic activity and T cell suppression are lipoxin-A4 (LXA4) dependent [109]. In addition, hAECs modulated the pulmonary inflammatory response to ventilation in preterm neonatal lambs and reduced acute lung injury [110]. Other studies reported that leukocyte infiltration of the lungs was not reduced by hAECs; however, the levels of inflammatory cytokines were reduced in intrauterine inflammation-induced lung injury [111] and in hyperoxia-induced neonatal lung injury [112]. Moreover, hAECs reduced the number of macrophages, dendritic cells, and natural killer cells and improved the tissue-to-airspace ratio and septal crest density in neonatal lung injury in a dose-dependent manner, regardless of the route of administration [113]. In early Achilles tendon defects, hAECs inhibited inflammatory cell infiltration, activated M2 macrophage subpopulation recruitment, and accelerated blood vessel and extracellular matrix remodeling by secreting immunoregulatory factors [68]. hAECs restored ovarian function by directly upregulating Treg cells in the spleen and reducing the inflammatory reaction in injured ovaries by modulating the polarization and function of macrophages in a paracrine manner [114]. In mice with experimental autoimmune thyroiditis (EAT) and systemic lupus erythematosus (SLE), hAECs prevented lymphocyte infiltration into the thyroid and improved thyroid follicular function by reducing the Th17/Treg cell ratio and increasing the proportion of B10 cells [115]. In addition, grafted hAECs accelerated diabetic wound healing and granulation tissue formation, partially by inducing differentiation of macrophages toward an M2 phenotype and promoting neovascularization in a paracrine manner [116]. In liver injury, hAECs induced the differentiation of macrophages toward an M2 phenotype, which was associated with a reduction in established hepatic fibrosis [117].

**Table 4:** Immunomodulatory function of hAECs in different diseases

Diseases/focuses	Transplantation method/dose	Species	Outcome	Repair mechanism	References
Ischemic stroke	Tail vein injection (1 × 10^6^ hAECs); saphenous vein injection (5 × 10^6^ hAECs)	Mice, marmosets	Reducing brain infarcted volume and functional deficits; promoting long-term functional recovery	Inhibiting apoptosis and inflammation; modulating immunosuppression	[70]
Intracerebral hemorrhage	Injection of cortex (1 × 10^6^ hAECs)	Rats	Reducing brain edema; ameliorating the neurologic deficits	Suppressing the activation of microglia; reducing the inflammatory response	[101]
Perinatal brain injury	Intravenously (1 × 10^5^ hAECs)	Mice	Reducing microglia apoptosis; increasing microglial phagocytic activity	Modulating microglia via releasing trophic factors	[102]
Fetal brain injury	Injection of brachial artery catheter (6 × 10^6^ hAECs)	Ewes	Reducing white matter injury; mitigating associated brain injury	Inhibiting inflammation and apoptosis; reducing the number of activated microglial cells	[103, 104]
Multiple sclerosis	Intravenously (2 × 10^6^ hAECs)	Mice	Reducing monocyte/macrophage infiltration and demyelination	Mediating immunosuppression via secreting TGF-β and PGE2; promoting Th2 cytokine shift	[105]
Lung injury	Intraperitoneally (4 × 10^6^ hAECs)	Mice	Decreasing neutrophil infiltration, fibrosis, collagen content; repairing lung function	Depending on the function of host macrophage	[106]
Lung injury	Intraperitoneally (4 × 10^6^ hAECs)	Mice	Reducing macrophage infiltration; increasing the number of M2 macrophage	Modulating macrophage polarization, migration, and phagocytosis via paracrine pathway	[107]
Lung injury	Intraperitoneally (4 × 10^6^ hAECs)	Mice	Mitigating lung inflammation and fibrosis	Tregs are required for hAEC-mediated macrophage polarization	[108]
Lung injury	Intraperitoneally (4 × 10^6^ hAECs)	Mice	Reducing pro-inflammatory immune cells; preventing lung injury	Mediating immunomodulation partly though LXA4	[109]
Preterm neonatal lung injury	Intratracheally (90 × 10^6^ hAECs)	Lambs	Modulating the pulmonary inflammatory response to ventilation; reducing acute lung injury	Immunomodulatory effects	[110]
Fetal lung injury	Fetal jugular vein injection (90 × 10^6^ hAECs); fetal intratracheal infusion (18 × 10^6^ hAECs)	Sheep	Attenuating the fetal pulmonary inflammatory response	Reducing inflammatory cytokines	[111]
Neonatal lung injury	Intraperitoneally (4.5 × 10^6^ hAECs)	Mice	Partially reducing hyperoxia-induced inflammation and structural lung damage	Attenuating inflammation	[112]
Neonatal lung injury	Intravenously; intratracheal infusion (5 × 10^4^; 7.5 × 10^4^; 1 × 10^5^ hAECs)	Mice	Improving the tissue-to-airspace ratio and the long-term of cardiorespiratory function	Reducing macrophages, dendritic cells, and natural killer cells	[113]
Achilles tendon injury	In situ filling (10 × 10^6^ hAECs)	Sheep	Inhibiting inflammatory cell infiltration; activating the M2 macrophage subpopulation	Regulating inflammatory and immunomodulatory response; accelerating blood vessel and ECM remodeling	[68]
Autoimmune ovarian disease	Intravenously (2 × 10^6^ hAECs)	Mice	Restoring ovarian function; upregulating Treg cells; reducing the inflammatory reaction	Modulating macrophage function by paracrine factors (TGF-β and MIF)	[114]
Experimental autoimmune thyroiditis; systemic lupus erythematosus	Intravenously (1.5 × 10^6^ hAECs); intravenously (1.5 × 10^6^ hAECs)	Mice	Preventing lymphocyte infiltration into the thyroid; improving the damage of thyroid follicular; reducing immunoglobulin profiles	Modulating the immune cell balance by downregulating the ratios of Th17/Treg cells; upregulating the proportion of B10 cells	[115]
Diabetic wound healing	Intradermally (1 × 10^6^ hAECs)	Mice	Promoting diabetic wound healing	Reducing inflammation and promoting neovascularization by paracrine pathway	[116]
Liver injury	Intravenously (2 × 10^6^ hAECs)	Mice	Reducing hepatic fibrosis	Inducing M2 macrophage phenotype	[117]

These immune remodeling processes, which are mediated by hAECs and soluble factors and extracellular vesicles secreted by hAECs, are of substantial importance to the regenerative process. Thus, hAECs have emerged as valid candidates for potential use in treating inflammatory and immune-based disorders.

### Clinical trials of hAEC transplantation

On the basis of the preclinical animal studies mentioned above, a series of clinical trials to assess the safety and effectiveness of hAEC transplantation in the treatment of various diseases have been registered at *http://ClinicalTrials.gov* and are being conducted (Table 5).

**Table 5:** Clinical trials of hAECs transplantation registered at *http://ClinicalTrials.gov*

	Study	Disease	Design	Start date	Status	Phase	Estimated enrollment	Intervention	***ClinicalTrials.gov*** identifier
1	Human Amniotic Epithelial Cell in Treatment of Refractory Severe Intrauterine Adhesion	Intrauterine adhesion	Safety and effectiveness	March 2018	Not yet recruiting	1	20	Uterine cavity infusion (100 million)	NCT03381807
2	Human Amniotic Epithelial Cells for Asherman’s Syndrome	Asherman’s syndrome	Safety and effectiveness	October 2017	Not yet recruiting	1	50	Biological amnion; biological amnion loaded with hAECs (100 million); intravenous infusion (100 million); intrauterine infusion (100 million); hydrogel loaded with hAECs (100 million)	NCT03223454
3	A Therapeutic Trial of Human Amniotic Epithelial Cells Transplantation for Primary Ovarian Insufficiency Patients	Primary ovarian insufficiency/premature ovarian failure/infertility	Safety and effectiveness	June 2020	Recruiting	1	36	Bilateral ovarian artery infusion (2 × 10^7^ cells)	NCT02912104
4	Human Amniotic Epithelial Cells Treatment for Ovarian Insufficiency	Premature ovarian failure	Safety and effectiveness	December 2017	Not yet recruiting	Not applicable	20	Minimally invasive implantation (200 million); intravenous infusion (100 million for 3 times)	NCT03207412
5	Human Amniotic Epithelial Cells for Treatment of Bronchial Fistula	Bronchial fistula	Therapeutic potential	October 2016	Recruiting	1	10	Endoscopic injection of hAECs to fistula (3–5 × 10^7^ cells)	NCT02959333
6	Effect of Human Amniotic Epithelial Cells on Children With Spastic Cerebral Palsy	Spastic cerebral palsy	Therapeutic potential	April 2017	Enrolling by invitation	1	10	Intrathecal injection	NCT03107975
7	Treatment of Non-union of Limb Fracture with Human Amniotic Epithelial Cells (hAECs)	Non-union fracture	Safety and efficacy	December 2017	Not yet recruiting	1/2	36	Transplant to non-union site (50 million)	NCT03031509
8	hAECs Are Preliminarily Applied in Allogeneic Hematopoietic Stem Cell Transplantation	Leukemia	Observational	July 2020	Recruiting	Not applicable	30	Unknown	NCT03759899
9	Human Amniotic Epithelial Cells Prevent Acute Graft-versus-host Disease After Hematopoietic Stem Cell Transplantation	Acute graft-versus-host disease	Safety and efficacy	July 2020	Recruiting	Not applicable	27	Infusion of hAECs (1 × 10^6^, 2 × 10^6^, 5 × 10^6^ cell/kg)	NCT03764228

Researchers conducted an early phase 1 clinical trial of hAEC transplantation in patients with intrauterine adhesion. Changes in the endometrial thickness, menstrual blood volume, and pregnancy rate will be observed to evaluate the safety and effectiveness of hAECs for treating intrauterine adhesion (NCT03381807). Another similar study was designed and conducted by using different interventional treatments with amnion, amnion loaded with hAECs, intravenous or intrauterine infusion of hAECs, and hydrogel loaded with hAECs to observe the therapeutic safety and effectiveness of hAECs in patients with intrauterine adhesion (NCT03223454).

Our research team designed and conducted a phase 1 clinical trial to evaluate the safety and effectiveness of bilateral ovarian artery infusion of hAECs into patients with POF. To date, we have recruited subjects and completed two trials of hAEC treatment in POF patients, and the results showed that hAEC transplantation increased the levels of estrogen and AMH, decreased FSH levels, and alleviated clinical symptoms (NCT02912104). Another clinical study of the application of hAECs to treat POF was also conducted via minimally invasive implantation. The outcomes were measured, including the ovarian volume and hormone levels (NCT03207412).

Investigators performed endoscopic injection of hAECs into bronchial fistula and observed the recovery of bronchial fistula and the resulting systemic reactions (NCT02959333). To evaluate the therapeutic potential of intrathecal hAECs in children with spastic cerebral palsy, functional status and spasticity were evaluated using the modified Ashworth scale (MAS) (NCT03107975). Phase 1/2 clinical trials were designed, in which hAECs were transplanted after debridement, and the efficacy and safety were evaluated for the treatment of non-union fractures (NCT03031509). In allogeneic hematopoietic stem cell transplantation (allo-HSCT) for the treatment of leukemia, researchers tried to develop clinically applicable hAEC products and to evaluate their preliminary application in allo-HSCT (NCT03759899). Additionally, a dose escalation study evaluating the safety and efficacy of hAECs in preventing acute graft-versus-host disease after HSCT was conducted (NCT03764228).

In addition, a clinical trial registered by Sievert et al. in the Australian and New Zealand Clinical Trails Registry (ACTRN12616000437460) was conducted to evaluate the safety of intravenously administered hAECs for the treatment of liver fibrosis. In this phase 1 clinical trial, patients who received hAEC transplantation were closely monitored in the first 24 h postinfusion, and long-term follow-up included standard liver tests, transient electrography, and hepatic ultrasound [118]. Another study reported that allogeneic hAECs could be safely infused into babies with established bronchopulmonary dysplasia (BPD), and there were no adverse events related to cell administration [119]. Further clinical trials (ACTRN12618000920291) were registered and conducted to evaluate the effectiveness of intravenous hAEC infusion, including a trial to assess the cytokine profile, respiratory outcomes, and neonatal morbidity of infants [120]. In addition, a phase I clinical hAEC therapy of ischemic stroke (ACTRN12618000076279) was designed to determine the maximal tolerated dose (MTD) and assess cell safety. Fifteen stroke patients will be recruited and injected with hAECs by intravenous infusion. Safety and efficacy will be assessed by imaging and immunological assays [121]. These clinical trials will help to determine the safety and clinical benefits of hAEC-based therapy.

### Prospects for hAEC-based therapy

The plasticity and therapeutic properties of hAECs are summarized in this review. Enhancing the repair potential of hAECs and achieving precise treatment need to be further considered.

To refine the manufacturing of hAECs and to maximize their capacity to promote functional recovery, there is an increasing need to improve our understanding of the biology and repair mechanism of hAECs. In consideration of special biological characteristics, primary hAECs have been widely used to repair damaged tissues in animal models and preclinical research. However, there are still some bottlenecks, including isolation protocols, EMT process, cell heterogeneity, and Measurement methods which hinder the clinical transformation of hAECs [122, 123]. Therefore, establishing a reasonable and optimal isolation protocol and agreeing upon strict definitions for hAECs will be necessary for their future application in tissue regeneration. Second, exploring new biology-guided approaches, including preconditioning and genetic manipulation, will facilitate their self-renewal and therapeutic properties of hAECs. For example, a recent study reported that vitamin C promoted the proliferation, migration, and self-renewal of hAECs. Furthermore, hAECs treated with vitamin C showed increased survival after transplantation and secreted a greater amount of growth factors, which improved the therapeutic potential of hAECs in mice with POF [124]. The plant-derived bioactive compound verbenalin significantly increased gene expression in hAECs, contributing to the enhanced neural repair potential of hAECs for AD [125].

On the other hand, paracrine function has been regarded as the main underlying mechanism in hAECs that mediates the recovery of function and immunomodulation; however, many factors can affect the paracrine potential of hAECs. A study revealed that primary and expanded hAECs displayed different differentiation capacity, immunosuppressive property, and paracrine effect, which could be exploited for different cellular therapeutic applications [29]. To enhance the repair potential and facilitate clinical application, some pretreatment approaches need to be further explored. A study showed that prolonged exposure of hAECs to the inflammatory cytokines interferon-gamma (INF-γ) and interleukin-beta (IL-1β) may result in enhanced expression and secretion of immunomodulatory molecules, which are important in treating immune-related diseases [126]. Furthermore, a wide variety of studies should be conducted to validate the potential of hAEC-exosomes, in which miRNAs and proteins are contained as cargo, and to optimize culture conditions to obtain the optimal secretome with therapeutic value for application.

Currently, combining biomaterials with hAECs has become a new approach to prolong and strengthen their beneficial effects. Studies have demonstrated that biomaterial scaffolds could contribute to positively modulating the inflammatory response in the tissue and stimulating tissue regeneration. For example, the implantation of hAECs loaded on hydroxyapatite/β-tricalcium phosphate scaffolds not only improved bone regeneration by direct participation but also reduced the early host immune response to the scaffolds [54]. Moreover, biomaterial scaffolds supported hAEC survival and differentiation after transplantation and provided a good microenvironment for tissue regeneration and functional recovery [127]. The latest study reported that poly(lactide-co-glycolide) (PLGA) could be used as a tendon biomimetic fleece for enhancing the differentiation and immunomodulation of transplanted hAECs in injured tendons [128]. Therefore, in vivo and long-term preclinical studies are needed to achieve translation from the bench to the bedside.

## Conclusions

Regenerative medicine is a broad field of medicine in which stem cells are used to regenerate the function of injured organs and tissues. hAECs are easy to isolate, have low immunogenicity, and lack ethical concerns; thus, hAECs have extremely important therapeutic potential in regenerative medicine. The current review helps to further explain the different mechanisms of action for hAEC-based cell therapy in treating various diseases. Importantly, the effects of hAEC paracrine function on the injured tissue microenvironment and the maintenance of balance of immunosuppression in recipients were crucial to the process of functional recovery. In the future, enhancing the therapeutic potential and developing new clinical protocols are needed for the application of hAEC-based strategies in regenerative medicine.

## Data Availability

Not applicable

## Abbreviations

3D: Three-dimensional
AD: Alzheimer’s disease
AGN: Angiogenin
ALP: Alkaline phosphatase
AOD: Autoimmune ovarian disease
BDNF: Brain-derived neurotrophic factor
bFGF: Basic fibroblast growth factor
BMP: Bone morphogenetic protein
BPD: Bronchopulmonary dysplasia
DA: Dopamine
EAE: Experimental autoimmune encephalomyelitis
EAT: Experimental autoimmune thyroiditis
EGF: Epidermal growth factor
EMT: Epithelial to mesenchymal transition
GMP: Good manufacturing procedure
hAEC-CM: Conditioned media of hAECs
hAEC-exosomes: hAEC-derived exosomes
hAECs: Human amniotic epithelial cells
hCECs: Human corneal epithelial cells
HGF: Hepatocyte growth factor
HLA: Human leukocyte antigens
HSCT: Hematopoietic stem cell transplantation
HSECs: Hepatic sinusoidal endothelial cells
ICH: Intracerebral hemorrhage
IGF: Insulin-like growth factor
IL-10: Interleukin-10
IL-1β: Interleukin-1beta
IL-5: Interleukin-5
INF-γ: Interferon-gamma
ISCs: Insulin secreting cells
LPS: Lipopolysaccharide
LXA4: Lipoxin-A4
MAS: Modified Ashworth scale
miRNAs: MicroRNAs
MMP: Matrix metalloproteinase
MTD: Maximal tolerated dose
NASH: Non-alcoholic steatohepatitis
NGF: Nerve growth factor
NT-3: Neurotrophin-3
OCT-4: Octamer-binding transcription factor-4
OPN: Osteopontin
PBMCs: Peripheral blood mononuclear cells
PD: Parkinson’s disease
PDGFB: Platelet-derived growth factor B
PDX1: Pancreatic and duodenal homeobox-1
PGE2: Prostaglandin E2
POF: Premature ovarian failure
SAGM: Small airway growth medium
SCs: Schwann-like cells
S-ihCECs: Immortalized human corneal epithelial cells
SLE: Systemic lupus erythematosus
SSEA-3/4: Stage-specific embryonic antigen-3/4
SSM: Substitute serum medium
SSS: Serum substitute supplement
TGF-β: TGF-beta
TRA: Tumor rejection antigen
VEGF: Vascular endothelial growth factor
